# The Potential Immunomodulatory Effect of *Bifidobacterium longum* subsp. *longum* BB536 on Healthy Adults through Plasmacytoid Dendritic Cell Activation in the Peripheral Blood

**DOI:** 10.3390/nu16010042

**Published:** 2023-12-21

**Authors:** Yiran Li, Satoshi Arai, Kumiko Kato, Sadahiro Iwabuchi, Noriyuki Iwabuchi, Natsumi Muto, Hideki Motobayashi, Shukuko Ebihara, Miyuki Tanaka, Shinichi Hashimoto

**Affiliations:** 1 Innovative Research Institute, R&D Division, Morinaga Milk Industry Co., Ltd., 5-1-83, Higashihara, Zama 252-8583, Kanagawa, Japan; 2 Department of Molecular Pathophysiology, Wakayama Medical University, 811-1 Kimiidera, Wakayama 641-8509, Wakayama, Japan; 3 Second Department of Surgery, Wakayama Medical University, 811-1 Kimiidera, Wakayama 641-8509, Wakayama, Japan; 4 Chiyoda Paramedical Care Clinic, Daiwa Building 2F, 3-3-10 Nihonbashi Hongokucho, Chuo-ku, Tokyo 103-0021, Japan

**Keywords:** *Bifidobacterium longum*, immunomodulatory, plasmacytoid dendritic cell, probiotics

## Abstract

The interaction between the gut microbiota and the host can influence the host’s immune system. *Bifidobacterium*, a commensal genus of gut bacteria, seems to have positive effects on host health. Our previous clinical research showed that *B. longum* subsp. *longum* BB536 enhanced innate and adaptive immune responses in elderly individuals with a lower grade of immunity, but the immunomodulatory mechanism is still unclear. In this study, dendritic cell (DC) surface markers in peripheral blood mononuclear cells isolated from healthy individuals were evaluated through coculture with heat-killed BB536. DC markers, innate immune activity and cytokine levels in plasma were also evaluated by a randomized, double-blind, placebo-controlled, parallel-group study (UMIN000045564) with 4 weeks of continuous live BB536 intake. BB536 significantly increased the expression of CD86 and HLA-DR on plasmacytoid DCs (pDCs) in vitro. Compared to placebo (*n* = 48), a significant increase in the expression of CD86 on peripheral pDCs was detected at week 4 of live BB536 intake (*n* = 49; 1 × 10^10^ CFU/day). Furthermore, coculture with hk-BB536 significantly increased the *IFNγ* expression level and demonstrated trends of increased *IFNα1* and *IFNβ* expression. These findings suggest that consumption of BB536 has potential immunomodulatory effects on healthy individuals through the activation of peripheral pDCs.

## 1. Introduction

The intricate interplay between the human immune system and the microbiota has gained significant attention in recent years. The human gut microbiota plays a crucial role in shaping immune responses and maintaining overall health [1]. Probiotics, defined as live microorganisms that confer health benefits to the host when administered in adequate amounts, have emerged as promising modulators of immune responses [2].

Bifidobacteria are abundant members of the human gut microbiota, particularly in infants, where they constitute a significant proportion of the early colonizers [3]. Among its diverse microbial inhabitants, Bifidobacteria have gained considerable attention for their potential immunomodulatory effects. These beneficial bacteria have demonstrated the ability to interact with the host’s immune system, influencing its homeostasis [4,5].

*Bifidobacterium longum* BB536, which was isolated from an infant, is a clinically effective and well-established multifunctional probiotic. It has a long history of human use in alleviating intestinal disorders, relieving pollen hypersensitivities, maintaining immunity, and preventing pathogenic infections [6]. Administration of *B. longum* BB536 has significantly reduced the duration of upper respiratory illnesses caused by the common cold, particularly the duration of sore throat, compared to the placebo group, in preschool children (aged 2–6 years old) [7]. In elderly individuals, taking *B. longum* BB536 significantly reduced the frequency of fever, inhibited the decline in natural killer (NK) cell activity and neutrophil phagocytic activity in peripheral blood, and increased antibody titers in the body [8]. These results imply that *B. longum* BB536 has the potential to enhance host immunity and shorten the duration of respiratory illnesses. However, the specific interactions of BB536 with the human gut immune system are unclear.

Since more than 70% of immune cells are present in the gut, the interaction between gut bacteria and intestinal epithelial cells is increasingly believed to be critical in influencing the host immune system [9]. Dendritic cells (DCs) can be broadly categorized into conventional DCs and plasmacytoid DCs (pDCs) following maturation [10]. They are primarily located within secondary lymphoid organs, particularly in the lung and intestinal mucosal environments [11,12], and are a very plastic cell population of antigen-presenting cells that hold a central position in innate and adaptive immunity. pDCs specialize in recognizing viral infections and producing significant levels of type I interferons (IFNs), which are essential cytokines for downstream stimulation and antiviral defense. pDCs can promote the activation and differentiation of T cells by direct interaction and antigen presentation [13,14], and interact with B cells to promote their differentiation and enhance antibody secretion by providing costimulatory signals and cytokines [15,16]. pDCs can also enhance the cytotoxic function of NK cells by type I IFN to reinforce the immune response against pathogens [17,18,19]. Overall, the stimulation by pDCs has broad effects on various immune cells, leading to a coordinated and effective immune response against pathogenic infection. Although probiotics can induce the activation of pDCs [20,21], the interaction of the genus *Bifidobacterium* with the human immune system has not been studied. We hypothesized that oral administration of BB536 induces the host immune response by interacting with pDCs in the gut. Therefore, we aimed to investigate whether BB536 could induce the activation of pDCs in vitro and in a clinical trial.

## 2. Materials and Methods

### 2.1. In Vitro Analysis

This study adhered to the current revision of the Declaration of Helsinki (2013) and the ethical guidelines for medical and health research involving human subjects (2015). The research protocol and informed consent form were approved by the Institutional Review Board (IRB) of Wakayama Medical University, Japan (Approval No. 3345). Written informed consent was obtained from healthy adult donors.

#### 2.1.1. Preparation of Peripheral-Blood Mononuclear Cells (PBMCs)

Twenty milliliters of peripheral blood were collected from each participant. The blood was used for mononuclear cell isolation as soon as possible using a Lymphoprep™ Tube (Serumwerk, Bernburg, Germany) following the instructions provided. The red blood cells were removed by treating them with an ammonium chloride solution (eBioscience, San Diego, CA, USA) for 10 min at 25 °C. After hemolysis, the cells were washed twice with cold D-PBS (Fujifilm, Tokyo, Japan), counted, and used as PBMCs for the experiments.

#### 2.1.2. Coculture of PBMCs and Heat-Killed *B. longum* BB536

*Bifidobacterium longum* subsp. *longum* BB536 (BB536) was obtained from stock cultures maintained at the Morinaga Culture Collection (Morinaga Milk Industry Co., Ltd., Tokyo, Japan). The bacteria were cultured for 16 h at 37 °C in MRS broth (DIFCO, Mich., Detroit, MI, USA) with 0.05% L-cysteine. Afterward, bacteria were collected and washed with sterile distilled water three times to completely remove the medium component. The bacteria were treated at 95 °C for 30 min, counted, and suspended in RPMI-1640 medium. PBMCs were seeded at 5 × 10^5^ cells/well in a 24-well plate with 1 × 10^6^ cells/mL heat-killed BB536 (hk-BB536) or Cpg-ODN 2216 (1 μM) (InvivoGen, San Diego, CA, USA). A well with no addition was designed as the control. The cocultivation was carried out at 37 °C under 5% CO_2_ and humid conditions.

#### 2.1.3. Fluorescence Activated Cell Sorter (FACS) Analysis

After 24 h of cocultivation, PBMCs were stained with FITC-labeled anti-human CD304 (clone No. U21-1283), PE-Cy7-labeled anti-human CD123 (clone No. 7G3), APC-labeled anti-human CD86 (clone No. 2331), and PE-labeled anti-human HLA-DR (clone No. G46-6) (BD, Franklin Lakes, NJ, USA) following treatment with Horizon Fixable Viability Stain 780 (BD, Franklin Lakes, NJ, USA) and Human BD Fc Block (BD, Franklin Lakes, NJ, USA) treatment. The cells were then washed twice with Stain Buffer and fixed with Cytofix Buffer (BD, Franklin Lakes, NJ, USA) for FACS analysis. Flow cytometry was used for analysis of surface markers of pDCs by selecting pDCs defined as CD123^+^CD304^+^ cells. CD86^+^HLA-DR^+^ pDCs were gated to assess the activation of surface markers. The data were processed and obtained using FlowJo ver. 7.6 (Tree Star, Ashland, OR, USA).

#### 2.1.4. Gene Expression Analysis

Total RNA was extracted using a NucleoSpin^®^ RNA Plus kit (Takara, Kusatsu, Japan) after 4 h of cocultivation. Complementary DNA was prepared using PrimeScript RT Master Mix (Takara, Japan) following the manufacturer’s protocol. qRT–PCR was performed using SYBR Premix Ex Taq (Takara, Japan) in a 7500 FAST Real-time PCR System (Applied Biosystems, Waltham, MA, USA). The amplification program consisted of an initial hold at 95 °C for 30 s, followed by 40 cycles of 95 °C for 3 s, and 60 °C for 30 s. The sequences of the primers are listed in Table 1. The 2^−ΔΔCt^ method was applied to calculate the relative gene expression, which was normalized to that of the housekeeping gene *β-ACTIN*.

### 2.2. Clinical Study

#### 2.2.1. Participants

One hundred participants were Japanese adults aged ≥20 years and <65 years who provided written informed consent. Participants with any other chronic or acute diseases, drug or severe food allergy, those using any probiotic supplement or yogurt, individuals engaging in excessive alcoholic drinking, those who were vaccinated for influenza or COVID-19 within one month before screening, and those with any vaccination plans during the study were excluded. Additionally, participants who were deemed unsuitable for the study by the principal investigator or sub-investigator for any reason were also excluded.

#### 2.2.2. Study Design and Intervention

This study was conducted as a randomized, double-blind, placebo-controlled parallel-group study approved by the Institutional Review Board of Chiyoda Paramedical Care Clinic (Tokyo, Japan). The study protocol was registered in the UMIN Clinical Trials Registry (UMIN000045564). One hundred participants were randomly assigned to the placebo or BB536 group based on the pDC and mDC FACS results, sex, BMI, and blood and urine parameters before administration. Randomized participants received lyophilized powder containing *B. longum* subsp. *longum* BB536 at approximately 10 billion colony-forming units with maltodextrin daily or placebo powder containing maltodextrin only and were orally administered sterilized milk for 4 weeks. Blood samples were collected before and after the intervention.

#### 2.2.3. FACS Analysis

Eighteen milliliters of peripheral blood were collected for PBMCs preparation. They were isolated as described in Section 2.1.1. PBMCs were stained with FITC-labeled anti-human CD304, PE-Cy7-labeled anti-human CD123, APC-labeled anti-human CD86, and PE-labeled anti-human HLA-DR for pDCs and were stained with PE-Cy7-labeled anti-human CD11c, FITC-labeled anti-human Lin1, APC-labeled anti-human CD86, and PE-labeled anti-human HLA-DR (BD, Franklin Lakes, NJ, USA) for mDCs, following treatment with Horizon Fixable Viability Stain 780 (BD, Franklin Lakes, NJ, USA) and Human BD Fc Block (BD, Franklin Lakes, NJ, USA) treatment. The cells were then washed twice with Stain Buffer and fixed with Cytofix Buffer (BD, Franklin Lakes, NJ, USA) for FACS analysis. The data were processed and obtained using FlowJo ver. 7.6 (Tree Star, Ashland, OR, USA).

#### 2.2.4. Measurements of NK Cell Activity, Neutrophilic Phagocytosis, and Bactericidal Activities

Nine milliliters of peripheral blood were collected for the measurement of NK cell activity, which was conducted by SRL Inc. (Tokyo, Japan) using the chromium-51 (^51^Cr) release method. Additionally, neutrophil phagocytosis and bactericidal activity were measured by LSI Medience Corporation (Tokyo, Japan).

#### 2.2.5. Evaluation of Immunological Responses to TLR Ligand of PBMCs Ex Vivo

The immunological responses to Cpg-ODN 2216 and R848 were evaluated as follows: PBMCs were seeded in 96-well plates at a density of 2 × 10^5^ cells/well and stimulated with Cpg-ODN 2216 (1 μM) or R848 (10 μg/mL) for 4 h. Afterward, total RNA was isolated, and qRT–PCR was performed as described in Section 2.1.4.

#### 2.2.6. Measurements of Cytokines in Plasma Isolated from Peripheral Blood

The plasma obtained from the PBMC isolation step was used for cytokine measurement. The concentrations of IFNα2a, IFNβ, IFNγ, IL-10, IL-12/IL-23p40, IL-12p70, IL-15, IL-23, and MCP-1 were measured using the U-PLEX Biomarker Group 1 (human) Assays (MSD Maryland, Rahway, NJ, USA) in duplicate samples. The measurements were performed according to the manufacturer’s protocol, and the data were collected using MESO Quick Plex SQ 120 (MSD Maryland, Rahway, NJ, USA). The data processing and concentration calculations were conducted using DISCOVERY WORKBENCH 4.0 (MSD Maryland, Rahway, NJ, USA).

### 2.3. Statistics

All data are presented as mean and standard error. Statistical analysis was conducted using SPSS IBM version 26 (IBM Corp., Armonk, NY, USA). To assess the statistical significance of the gene expression differences between experimental groups, their ΔCt values were compared. For the geometric mean of DC markers, Tukey’s multiple comparison test was performed for statistical analysis of coculture data in vitro. Additionally, an analysis of covariance (ANCOVA) was conducted, setting the test food group as the explanatory variable and the baseline values as covariates. The Shapiro–Wilk test for normality was employed. The intragroup changes compared to baseline were assessed using a paired *t* test since the data conform to a normal distribution. The nonparametric Mann–Whitney U test was utilized to compare group difference in NK activity, neutrophilic phagocytosis and bactericidal activities, IFN gene expression in PBMCs and cytokine concentrations in plasma since the null hypothesis of a normal distribution was rejected according to the Shapiro–Wilk test. Graphs were generated using Prism 9.0.

## 3. Results

### 3.1. Heat-Killed BB536 Activated the Surface Markers of pDCs in PBMCs In Vitro

**Figure 1:**
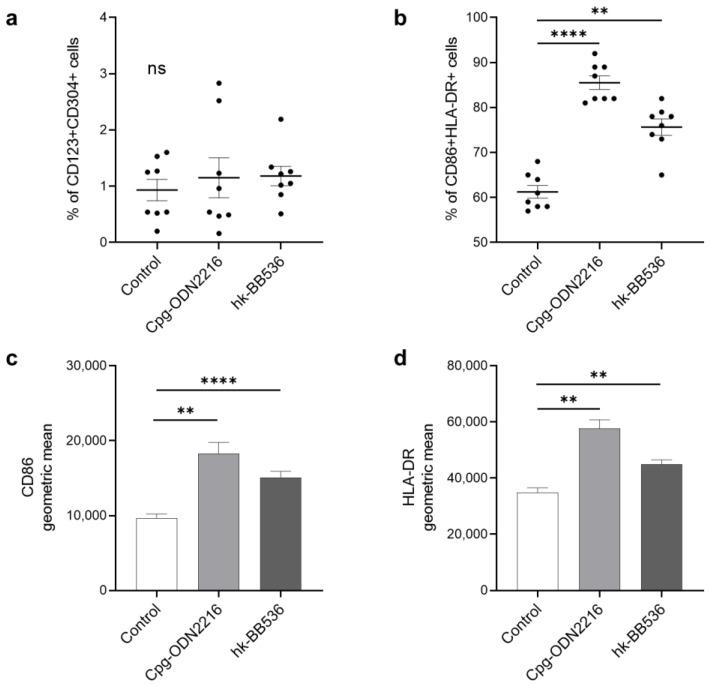
Effects of Cpg-ODN 2216 and hk-BB536 on the activation of pDC markers. (**a**) The percentages of CD123^+^CD304^+^ cells in total live cells; (**b**) the percentages of CD86^+^HLA-DR^+^ cells in pDCs; (**c**) the geometric means of CD86 activation in total pDCs; (**d**) the geometric means of HLA-DR activation in total pDCs. All the data are expressed as the mean ± SE (*n* = 8). **, *p* < 0.01 and ****, *p* < 0.0001 significant difference vs. the control; ns: no significant difference.

In this study, the results of flow cytometry analysis showed that cocultivation of Cpg-ODN 2216 or hk-BB536 did not affect the percentage of CD123^+^CD304^+^ cells among total live cells (Figure 1a). Cpg-ODN 2216, a potent TLR9 ligand, strongly induced the expression of the surface markers CD86 (*p* < 0.01) and HLA-DR (*p* < 0.01) (Figure 1c,d). After 24 h of cocultivation of PBMCs with hk-BB536, the ratio of CD86^+^HLA-DR^+^ to total pDCs significantly increased (Figure 1b). Hk-BB536 also upregulated the surface markers CD86 (*p* < 0.0001) and HLA-DR (*p* < 0.01) on pDCs compared with those of the control (Figure 1c,d).

### 3.2. Effect of Hk-BB536 on Interferon Gene Expression in PBMCs In Vitro

**Figure 2:**
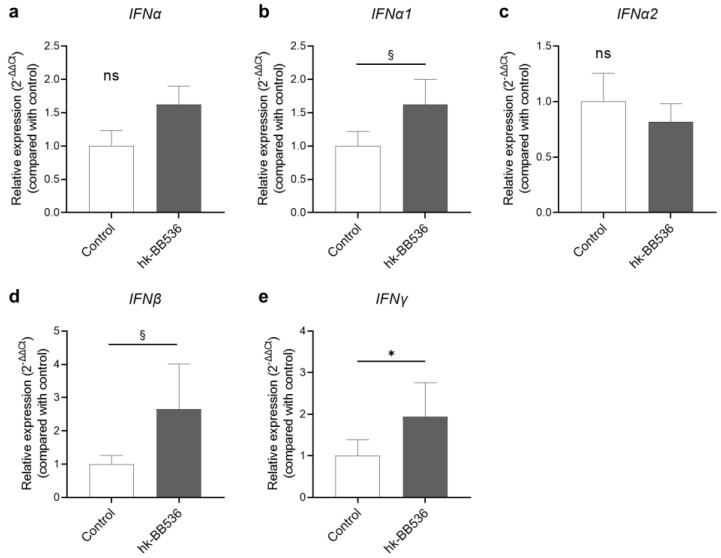
Effects of hk-BB536 on the gene expression levels in PBMCs by qRT–PCR analysis. (**a**) *IFNα*; (**b**) *IFNα1*; (**c**) *IFNα2*; (**d**) *IFNβ*; (**e**) *IFNγ*. All the data are expressed as mean ± SE (*n* = 8). *, *p* < 0.05 and §, 0.5 ≤ *p* < 0.1 vs. the control; ns: no significant difference.

To further determine whether hk-BB536 affects the immune response of PBMCs, the relative gene expression levels of IFN genes, IFNα, IFNα1, IFNα2, IFNβ, and IFNγ, in PBMCs were analyzed by qRT–PCR after cocultivation with hk-BB536 or Cpg-ODN 2216 for 4 h. Cpg-ODN 2216 induced an extremely high expression of these genes compared with the control. Cocultivation with hk-BB536 significantly increased IFNγ expression (*p* < 0.05) (Figure 2e), and high trends were detected in the expression of IFNα1 and IFNβ (Figure 2b,d) in PBMCs compared with the control.

### 3.3. Initial Participant Characteristics in the Clinical Study

**Figure 3:**
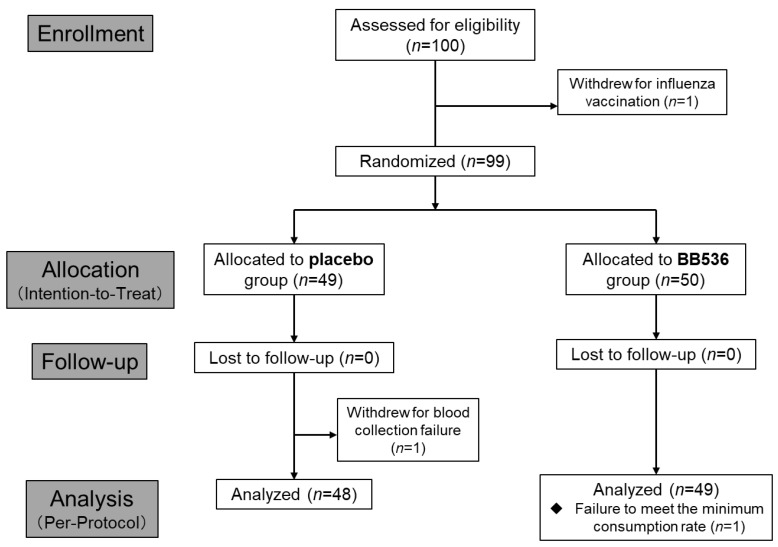
CONSORT flow chart for this randomized, double-blinded, placebo-controlled clinical trial.

A total of 100 healthy adults were asked to participate in the study, and one participant withdrew from the study due to personal reasons. Blood samples collected from the participants at the screen visit were used for pDC surface marker CD86 and HLA-DR analysis and safety parameter testing. The results of surface marker activity on pDCs were considered a priority factor for randomization. One participant withdrew from the trial due to personal vaccination plan. The remaining 99 participants were randomized into two groups receiving either live BB536 (1 × 10^10^ CFU/day; *n* = 50) or placebo (without BB536; *n* = 49). One participant in the placebo group withdrew due to failure in blood collection for physical reasons. Additionally, one participant was excluded from the per-protocol analysis due to failure to meet the minimum consumption rate (80%) in the BB536 group (Figure 3). The baseline characteristics of the analyzed participants (intention-to-treat population) in the two groups are presented in Table 1. No significant differences between the groups were found (Table 2), and no significant clinical adverse effects were reported during the intervention period.

### 3.4. Effects of BB536 Intake on DC Activity In Vivo

**Figure 4:**
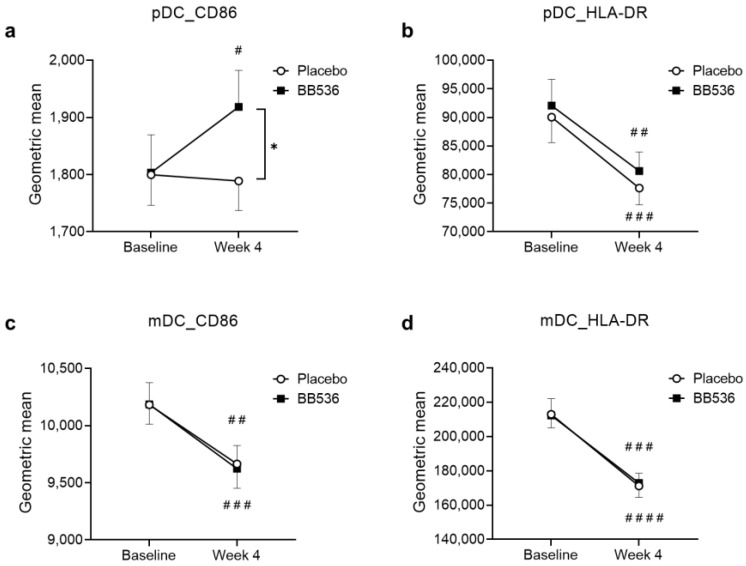
Effects of BB536 administration on activity changes in pDCs and mDCs (as measured by surface markers) in vivo, comparison between the placebo and BB536 groups before and after the 4-week intake period. (**a**) CD86 activities on pDCs; (**b**) HLA-DR activities on pDCs; (**c**) CD86 activities on mDCs; (**d**) HLA-DR activities on mDCs. All data are mean ± SE. *, *p* < 0.05 between the two groups; #, *p* < 0.05, ##, *p* < 0.01, ###, *p* < 0.001, and ####, *p* < 0.0001 within each group before vs. after the intervention.

We detected that hk-BB536 showed stimulatory activity on pDCs in vitro. We evaluated DC activity, including the surface markers CD86 and HLA-DR, on pDCs and mDCs, respectively, in PBMCs before and after 4 weeks of BB536 administration in our randomized, double-blind, placebo-controlled clinical study. There were no significant differences between the BB536 and placebo groups at baseline for any of the markers. After the 4-week intervention, a significant increase in CD86 activity on pDCs was observed in the BB536 group (*p* < 0.05), but no change was observed in the placebo group. At week 4, the expression of CD86 on pDCs was significantly higher in the BB536 group than in the placebo group (*p* < 0.05) (Figure 4a). Although there were significant decreases in the surface markers HLA-DR on pDCs and CD86 and HLA-DR on mDCs in both the BB536 and placebo groups, no significant intergroup change was observed (Figure 4b–d). 

### 3.5. Effects of BB536 Intake on NK Activity, Neutrophil Phagocytic Activity, and Bactericidal Activity In Vivo

**Figure 5:**
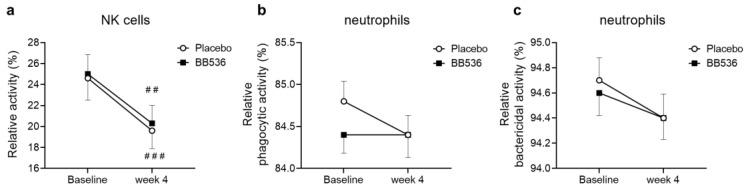
Relative NK cell activity (**a**) and neutrophil phagocytic (**b**) and bactericidal activities (**c**) in PBMCs before and after 4 weeks of placebo and BB536 intake. All data are mean ± SE. ##, *p* < 0.01, and ###, *p* < 0.001 within groups before vs. after the intervention.

We investigated NK activity and neutrophil phagocytic and bactericidal activities in the peripheral blood of participants in the placebo and BB536 groups before and after 4 weeks of intake. Compared to baseline, significant decreases were observed in NK activity in both the placebo (*p* < 0.001) and BB536 groups (*p* < 0.01) (Figure 5a). There was no significant difference between the placebo and BB536 groups after 4 weeks of intake (Figure 5).

### 3.6. Effects of BB536 Intake on the IFN Response Ex Vivo

To evaluate the immune response of PBMCs in peripheral blood, the relative expression levels of the IFNα, IFNα1, IFNα2, IFNβ, and IFNγ genes in PBMCs were quantitatively analyzed by qRT–PCR after 4 h of stimulation with R848 (Table 3) or Cpg-ODN 2216 (Table 4). No significant difference was found between placebo and BB536 at baseline or week 4; however, the −ΔΔCt change in IFNα1 in the BB536 group before and after intake was significantly lower than that in the placebo group with Cpg-ODN2216 stimulation ex vivo (Table 4).

### 3.7. Effects of BB536 Intake on the IFN Response in Plasma Ex Vivo

To accurately determine the cytokines in plasma, the cytokine concentrations of IFNα2a, IFNβ, IFNγ, IL-10, IL-12/IL-23p40, IL-12p70, IL-15, IL-23, and MCP-1 were evaluated using the ECL-based U-PLEX assay method because of the high sensitivity of this immunoassay in detecting the trace amount of target molecules in the sample. Even so, the measurement values of IFNα2a, IFNβ, IFNγ, IL-10, IL-12p70, and IL-23 were below the limit of quantitation according to the manufacturer’s instructions. All of the measurement values of IL-12/IL-23p40, IL-15, and MCP-1 were within the range of values obtained from the standard curve, and these data were used for further statistical analysis.

**Figure 6:**
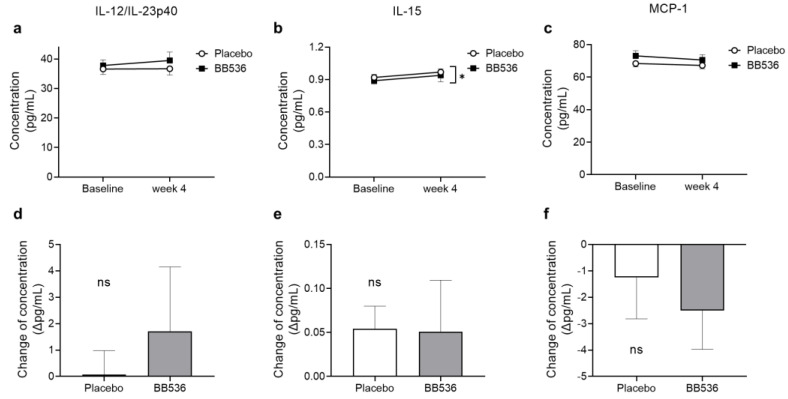
Cytokine concentrations in plasma and changes from baseline to after 4 weeks of placebo and BB536 intake in peripheral blood. (**a**) IL-12/IL-23p40 concentration in plasma; (**b**) IL-15 concentration in plasma; (**c**) MCP-1 concentration in plasma; (**d**) IL-12/IL-23p40 concentration change; (**e**) IL-15 concentration change; (**f**) MCP-1 concentration change. All data are mean ± SE. *, *p* < 0.05 between groups. ns: no significant difference.

As shown in Figure 6, there was no significant difference in IL-12/IL-23p40, or MCP-1 at baseline or at week 4 between the placebo and BB536 groups (Figure 6a,c). After 4 weeks of intake, the BB536 group showed significantly lower IL-15 than the placebo group (Figure 6b). Considering that the individual differences would impact the statistical results, the changes in cytokine concentration from baseline to after 4 weeks of intake were calculated, and no significant difference was observed in IL-12/IL-23p40, IL-15, or MCP-1 (Figure 6d–f).

## 4. Discussion

Intake of BB536 reportedly enhanced the immune response by maintaining NK activity and increasing IgA in serum in hospitalized elderly patients with enteral nutrition. BB536 supplementation also elevated the acquired immune response by increasing the antibody titer after A/H1N1 influenza vaccination [22]. In this study, we demonstrated that BB536 activated pDCs derived from human PBMCs and induced CD86 and HLA-DR marker expression in vitro. Our double-blinded, randomized clinical trial of healthy adults revealed that intake of BB536 increased CD86 expression on pDCs in PBMCs compared with placebo at week 4.

Of the gut immune cells, pDCs play a crucial role in defending the gastrointestinal tract against pathogens and maintaining host immune homeostasis [23,24]. The surface markers CD86 and HLA-DR on pDCs play important roles in the immune system. CD86 participates in immune regulation by engaging with its ligands CD28 and CTLA-4 on T cells to enhance T-cell activation, cytokine production, and proliferation to facilitate robust immune responses against pathogens or tumors [25,26], and HLA-DR can effectively present foreign antigens, such as pathogens or abnormal cells, to CD4^+^ T helper cells, initiating an adaptive immune response [27,28]. Wittmann et al. [29] used an anti-mouse PDCA-1 antibody to deplete pDCs in mice and found that *B. adolescentis* lost its protective function against *Yersinia enterocolitica* infection compared with the control and suggested that bifidobacteria activate the host immune response through pDC-mediated clearance of pathogens in the intestine. In this study, hk-BB536 significantly increased the expression of CD86 and HLA-DR on pDCs in PBMCs after cocultivation, and an increase in CD86 expression was also detected after 4 weeks of BB536 intake in our clinical study. Interestingly, no significant difference in the pDC ratio between hk-BB536 treatment and the control was observed. These results suggested that hk-BB536 might activate the immune response by elevating the expression of CD86 and HLA-DR in pDCs rather than stimulating pDC proliferation. However, no significant change in HLA-DR expression in pDCs was observed between the BB536 and placebo groups after the 4-week intervention. Compared with the direct bacteria–host cell interaction, the interaction following oral intake of BB536 seemed to exert only partial stimulation on peripheral pDCs through intestinal lymphatic organs.

Additionally, 4 h of cocultivation with hk-BB536 resulted in a significant increase in the expression level of *IFNγ*, and increasing trends were observed in the expression levels of *IFNα1* and *IFNβ*. IFNs, a group of signaling proteins circulating throughout the body, play a crucial role in the response to viral and other pathogenic infections [15,30]. In peripheral blood cells, pDCs act as essential sentinels and contribute mainly to the production of type I interferon [31] due to their constitutive expression of IRF7 at a high level [32]. However, Sugimura et al. [21] reported that IFNα production in the supernatant was undetectable by ELISA after heat-killed *Lactococcus lactis* JCM5805 stimulation of pDCs. In this study, although the S-PLEX assay, with very high detection sensitivity, was conducted to quantitate IFNα2a and IFNβ concentrations in the supernatant after 24 h of cocultivation, most of the readings were below the lower limit of detection. Unlike the pDC subpopulation in peripheral blood, gut pDCs isolated from Peyer’s patches exhibit reduced production of type I interferon compared to spleen-derived pDCs in mice. This distinction is attributed to the strict regulation of immune responses within the intestine, aimed at preventing excessive immune reactions against harmless antigens and commensal bacteria [33]. This may suggest an explanation as to why the induction of type I IFN gene expression occurred in cocultivation experiments yet no significant changes were observed in the clinical study. Compared to the acute immune response characterized by type I IFN (IFNα/β) production by peripheral pDCs during virus infection, gut pDCs tend to play an important role in homeostasis between tolerance and immunity to mucosal pathogens. It is reasonable to expect that the type I IFN production in vivo triggered by BB536 should be quite mild.

In previous studies, live BB536 bacteria highly induced the amount of IFNγ cocultivated with human PBMCs for 24 h [34]. IFNγ, as a vital cytokine, plays a crucial role in both innate and acquired immune responses, primarily activating macrophages and inducing the expression of MHC class II molecules. In this study, the IFNγ gene expression level was significantly induced by hk-BB536 in vitro (Figure 2e). The cytokine IL-12 is thought to be mainly produced by activated APCs, such as pDCs and macrophages, and can induce the production of IFNγ from NK cells and T cells [35]. The clinical study results showed that the IL-12 concentration in the plasma of the BB536 group rose more over the baseline level than it did in the placebo group, though there was no significant intergroup difference (Figure 6d). These results suggested that BB536 has potential effects on improving the host’s defense against pathogenic infections.

On the other hand, regarding the effects of BB536 on the level of the innate immune response, no obvious changes were detected in neutrophil phagocytic or bactericidal activities (Figure 5) or MCP-1 (Figure 6c). Considering the enormous variation among participants, although the IL-15 concentration in plasma significantly differed between BB536 and placebo at week 4, there was no difference in the IL-15 concentration before vs. after intake (Figure 6b,e). In addition, we noticed that many types of cytokines failed to be detected in plasma because they were below the limit of quantitation. There is a strong possibility that the subjects recruited for this clinical trial were all healthy individuals, and even though the clinical trial was conducted during the winter season amid the COVID-19 pandemic, the improvement of personal hygiene habits and mask wearing significantly reduced the chances of infection [36]. A rhythmic variation in NK cell activity has been reported in healthy hosts throughout the year, with a particularly dramatic increase occurring in the early stages of the winter season [37]. This increase in NK activity coincides with the start of the cold, dry weather of winter. The host innate immune response becomes more active in such conditions. Wearing masks greatly helps prevent the spread of viruses and hydrate the upper respiratory tract, such as the nasal mucosa and throat [38]. This practice potentially alleviates the excessive occurrence of innate immune responses, including NK cell and neutrophil activities. Previous clinical studies have demonstrated that orally administered probiotics could significantly elevate NK activity in peripheral blood. We noticed that these studies recruited individuals over 60 years old [39,40] or subjects with lower white blood cell counts than usual [41] and had longer intervention periods (≥8 weeks) [42], and their findings were consistent with our previous clinical outcomes [8,22]. No change in NK activity was detected after *Lactobacillus casei* Shirota intake in healthy male subjects (LcS, *n* = 34; Control, *n* = 34) over a 4-week intervention [43]. However, a significant increase in NK activity was observed in another clinical trial of 243 university athletes (LcS, *n* = 126; placebo, *n* = 117) after a 20-week ingestion period [44]. These previous findings suggest that the immune situation of subjects and the intervention period may result in different outcomes.

One limitation of this clinical study might be that significant fluctuations in immune-related clinical outcomes were difficult to observe because of the strong effects of protective measures taken during the COVID-19 pandemic and because their immune levels at baseline were within the normal range. Furthermore, the dosage and intervention period should also be explored further, and additional clinical studies without mask wearing are needed.

## 5. Conclusions

In conclusion, hk-BB536 significantly induced CD86 and HLA-DR expression on pDCs when cocultured with human PBMCs in vitro. A significant increase in CD86 in peripheral blood pDCs was confirmed after a 4-week oral intake of live BB536 compared to the placebo. Additionally, a significant increase in *IFNγ* expression and high trends in *IFNα1* and *IFNβ* expression were observed in vitro. These results suggest the potential of *B. longum* BB536 intake to improve the immune response of healthy adults through pDC activation.

## Figures and Tables

**Table 1:** Primers used in this study.

Gene	Forward Primer (5′-3′)	Reverse Primer (5′-3′)
*β-ACTIN*	GAGCGGGAAATCGTGCGTGACATT	TGCCCAGGAAGGAAGGCTGGAAGA
*IFNα*	GACCAGGAGACACGGAATGT	GATGTAATCCTTGCCGTCGT
*IFNα1*	GCAAGCCCAGAAGTATCTGC	ACTGGTTGCCATCAAACTCC
*IFNα2*	AAATACAGCCCTTGTGCCTGG	GGTGAGCTGGCATACGAATCA
*IFNβ*	AAGGCCAAGGAGTACAGTC	ATCTTCAGTTTCGGAGGTAA
*IFNγ*	TGACCAGAGCATCCAAAAGA	CTCTTCGACCTCGAAACAGC

**Table 2:** Baseline characteristics of the study participants (intention-to-treat population).

Characteristic	Placebo (*n* = 49)	BB536 (*n* = 50)	*p*-Value
Female (%)	28 (58.3%)	25 (50.0%)	0.548
Age (years)	45.9 ± 10.8	46.2 ± 10.5	0.903
BMI (kg/m^2^)	21.78 ± 2.1	21.43 ± 2.03	0.399
Smoker (%)	1 (2.08%)	3 (6.0%)	0.617
Sleep time (hour)	6.68 ± 0.75	6.66 ± 0.72	0.873
White blood cell (/μL)	5058.4 ± 1048.8	5149.2 ± 1198.8	0.689
Platelet count (×10^4^/μL)	27.29 ± 4.97	26.45 ± 4.57	0.385
Total serum protein (g/dL)	7.24 ± 0.35	7.29 ± 0.36	0.457
Albumin (g/dL)	4.48 ± 0.26	4.52 ± 0.27	0.425
γ-GTP (U/L)	21.6 ± 11.7	22.8 ± 11.3	0.613
HbA1c (%)	5.18 ± 0.19	5.26 ± 0.26	0.073
Triglyceride (mg/dL)	72.4 ± 39.6	68.3 ± 32.1	0.570
HDL-Cholesterol (mg/dL)	71.2 ± 14.7	68.9 ± 16.6	0.482
LDL-Cholesterol (mg/dL)	115.9 ± 21.9	117.0 ± 25.1	0.816
Total-Cholesterol (mg/dL)	204.6 ± 26.1	200.2 ± 24.9	0.402

Data are expressed as mean ± SE or n (%). The test values were evaluated by referring to the Japanese Reference Intervals published by the Japanese Committee for Clinical Laboratory Standards. BMI, body mass index; γ-GTP, γ-glutamyl transpeptidase; HbA1c, glycated hemoglobin; HDL, high-density lipoprotein; LDL, low-density lipoprotein.

**Table 3:** Relative expression levels of IFN genes with R848 stimulation ex vivo.

	Baseline ^1^			Week4		
	Placebo	BB536	*p-*Value	Placebo	BB536	*p-*Value
*IFNα*	4.671 ± 0.249	4.702 ± 0.237	0.943	4.883 ± 0.248	4.623 ± 0.188	0.209
*IFNα1*	4.808 ± 0.246	4.868 ± 0.236	0.937	5.128 ± 0.257	4.870 ± 0.185	0.218
*IFNα2*	5.700 ± 0.300	5.862 ± 0.292	0.901	5.898 ± 0.286	5.690 ± 0.228	0.421
*IFNβ*	4.980 ± 0.230	5.019 ± 0.218	0.811	5.468 ± 0.265	5.192 ± 0.207	0.268
*IFNγ*	4.979 ± 0.331	4.973 ± 0.236	0.914	6.249 ± 0.346	6.242 ± 0.191	0.736

^1^ All data are mean ± SE.

**Table 4:** Relative expression level of interferon genes with Cpg-ODN2216 stimulation ex vivo.

	Baseline ^1^			Week4		
	Placebo	BB536	*p*-Value	Placebo	BB536	*p*-Value
*IFNα*	4.671 ± 0.249	4.702 ± 0.237	0.943	4.883 ± 0.248	4.623 ± 0.188	0.209
*IFNα1*	4.808 ± 0.246	4.868 ± 0.236	0.937	5.128 ± 0.257	4.870 ± 0.185	0.218
*IFNα2*	5.700 ± 0.300	5.862 ± 0.292	0.901	5.898 ± 0.286	5.690 ± 0.228	0.421
*IFNβ*	4.980 ± 0.230	5.019 ± 0.218	0.811	5.468 ± 0.265	5.192 ± 0.207	0.268
*IFNγ*	4.979 ± 0.331	4.973 ± 0.236	0.914	6.249 ± 0.346	6.242 ± 0.191	0.736

^1^ All data are mean ± SE.

## Data Availability

Data are contained within the article.
